# 25(OH)D Concentration in Neonates, Infants, and Toddlers From Poland—Evaluation of Trends During Years 1981–2011

**DOI:** 10.3389/fendo.2018.00656

**Published:** 2018-11-08

**Authors:** Marek Wójcik, Maciej Jaworski, Pawel Pludowski

**Affiliations:** Department of Biochemistry, Radioimmunology and Experimental Medicine, The Children's Memorial Health Institute, Warsaw, Poland

**Keywords:** vitamin D, 25(OH)D, vitamin D deficiency, calcaemia, infants, toddlers

## Abstract

**Introduction:** The numerous evidence showing spectrum of vitamin D effects on human health resulted in both updates of vitamin D supplementation guidelines for general population and concerns on potential risk of hypercalcaemia. The aim of this study was to analyse trends in serum 25-hydroxyvitamin D concentration (25(OH)D) change over the 30 years of operation of a single pediatric diagnostic unit.

**Materials and methods:** Calcium-phosphate metabolism markers and 25(OH)D concentrations were analyzed in a group that consisted of newborns and infants commissioned for diagnostics due to suspected calcium-phosphate metabolic disturbances (*n* = 3,163; mean age 8.0 ± 3.0 months).

**Results:** 25(OH)D < 10 ng/ml was noted in 4.5% of patients (*n* = 163), 10–20 ng/ml in 14.7% (*n* = 465), 20–30 ng/ml in 23.9% (*n* = 756) and 30–50 ng/ml in 35.9% (*n* = 1,136). The mean 25(OH)D concentration in analyzed group was 37.5 ± 24.5 ng/ml. In patients with 25(OH)D concentration < 10 ng/ml a normal calcaemia (2.25–2.65 mmol/l) was noted in 83.4% cases (*n* = 136). Eighty one patients had 25(OH)D concentrations above 100 ng/ml with co-existing calcaemia in range of 2.6–4.38 mmol/l (mean Ca = 2.69 mmol/l). Hypocalcaemia (Ca < 2.25 mmol/l) was observed in 0.54%, (*n* = 17). 13.8% patients revealed calcium levels >2.65 mmol/l (*n* = 435). In general, the mean calcium-phosphate markers values were within the reference range for age. The highest mean 25(OH)D concentration of 51.8 ng/ml ± 38.8 was noted in years 1981–1999 (*n* = 305). The lowest mean 25(OH)D value was observed in years 2010–2011 (29.0 ng/ml ± 13.6; *n* = 412). The trend of decreasing 25(OH)D concentration during analyzed time period was significant (*r* = −0.29, *p* < 0.0001).

**Conclusions:** Eighty percentage of children aged 0–36 months had 25(OH)D concentration >20 ng/ml, however, during 3 decades a mean 25(OH)D concentrations trended significantly to decrease. A direct relationship between low 25(OH)D concentration and hypocalcaemia was not observed nor between high 25(OH)D concentration and hypercalcemia.

## Introduction

Over the last decade, the biologic activity of vitamin D and its metabolites in the human body has become the topic of many publications and intense discussions. The discoveries of the pleiotropic and multi-organ effects extended the knowledge on the basic role of vitamin D, i.e., the regulation of calcium and phosphate homeostasis. It was demonstrated that vitamin D deficiency is associated (or at least coincides to) with many diseases, such as neoplasms (1–5), autoimmune diseases (6–8), type 1 and type 2 diabetes (9–11), cardiovascular disease (12–14), and hypertension (15–17). Therefore, the interest in vitamin D deficiency and vitamin D supplementation as public health problems has increased. In light of increasing life expectancy and high prevalence of chronic diseases as well as negative changes in dietary habits and lifestyle, obtaining and maintaining optimal 25(OH)D concentrations has become an important aspect of health-oriented policies (18, 19).

Vitamin D has been used as an agent for the prevention and treatment of nutritional rickets, however, still majority of epidemiological studies indicate a significant deficiency of this vitamin (20–23). Therefore, it appeared crucial to establish satisfactory recommendations on vitamin D supplementation for the general population. The most commonly quoted position papers are the practice guidelines issued by the Endocrine Society in 2011 (24) and the supplementation recommendations published by the Institutes of Medicine (IOM) in 2010 (25). Vitamin D supplementation guidelines are available also for European countries, including Scandinavian countries (Denmark, Finland, Iceland, Norway and Sweden) (26) or Germany, Austria and Switzerland (27), Poland (28), and Central Europe (29). Despite differences between recommended vitamin D daily doses for general population, international scientific societies recognized 25(OH)D concentrations below 10 ng/ml as extremely low and reflecting severe vitamin D deficiency (20, 24, 25, 28–31). In light of the most recent studies, reference ranges have been changed and the minimum concentration considered as beneficial for health was set at 20 ng/ml or even 30 ng/ml, depending on the reference authority (24, 28, 29, 31). Taking into account the above mentioned criteria, vitamin D deficiency in Poland and in Europe seems to be a common issue (30, 32–34), at least in the age groups not protected by the nutritional rickets prevention programs (20).

However, regular vitamin D supplementation recommended by various scientific societies resulted in significantly increased use of vitamin D supplements by general population, and initiated discussions on vitamin D toxicity and the incidence of hypercalcemia. It has been emphasized that in some cases an excessive doses of cholecalciferol might increase the risk of hypercalcaemia, hypercalciuria, and nephrocalcinosis (35–37). British studies from the 1950s described cases of hypercalcaemia in infants, caused by the use of doses exceeding 4,000 IU a day (38). Some of these patients were of a phenotype later called Williams-Beuren syndrome (39), while others were decades later classified into a new disease entity of a genetic origin—idiopathic infantile hypercalcemia (IIH) (35–37, 40). Similar cases were also observed in Poland in the 1970–1980s (41, 42). Hypercalcaemia manifested itself after the supplementation with doses of 2,500–4,000 IU a day, as well as at loading doses up to 300,000 IU (35, 41, 42). For the above mentioned reasons, supplementation should be carried out carefully, and self-administration of vitamin D without medical supervision seems risky especially in patients with unrecognized hypersensitivity to vitamin D. The risk of vitamin D hypersensitivity is determined genetically (35–37, 43–45). According to the most recent study conducted in Poland the incidence of IIH in Polish population was estimated to be as high as 1:32,465 births (43).

Taking into account the historical aspect of vitamin D use in pediatric population before 2009, the available guidelines in Poland were not nationwide and were limited to nutritional rickets prevention in infants and in small children. Since the mid-1980s, the use of vitamin D loading doses of up to 300,000 IU in the prevention and treatment of nutritional rickets was discontinued in medical practice and the recommended preventive vitamin D intake in neonates and infants was set at a dose of 2,500 IU a day, and since the 1990s it was further lowered to 400–800 IU a day (46). The above approaches did not resulted in an increase of incidence of nutritional rickets in Poland and, most likely, the risk of potential adverse effects and health complications related to overdosing was reduced, at least in individuals with vitamin D hypersensitivity. Unfortunately, there are no extended studies from the 1980s and 1990s that had assessed vitamin D status in the population of neonates, infants and toddlers, especially in the context of vitamin D doses and related outcomes such as 25(OH)D concentrations and calcemia. Further, very few studies from that time had described vitamin D status in newborns from Poland (46) due to the limited availability of the quantitative measurement of 25(OH)D that was carried out only in the reference laboratory facilities. At 1980s and 1990s, the determination of 25(OH)D concentration in Polish children concerned only patients who were suspected of disorders of calcium and phosphate homeostasis and disturbed bone mineralization.

This study data might complement more recent evidence describing vitamin D status in the present populations of neonates, infants and toddlers, however the most of novel data was restricted to regional surveys from small territory and was done at a short period of time and/or was limited by inclusion criteria.

The population of pediatric patients from entire Poland, described below, includes an extensive historical period of the 1980s and 1990s as well as the first decade of the 21st Century. This is a unique group in terms of the period of time, relatively large number of pediatric patients, as well as their clinical and biochemical diversity. Our observations may at least in part uncover different aspects of vitamin D supplementation, vitamin D status and calcemia in the population of the youngest children in the so-far poorly described periods of time. An overview of historical data showing 25(OH)D concentrations and calcemia may add information to discussion on potential trends that occurred over the 30 years as well as give some insights on the relationship between vitamin D status and calcemia. This study was aimed to evaluate 25(OH)D concentration data assayed in newborns, infants and toddlers that were commissioned in the years 1981–2011 to the single diagnostic unit for the evaluation of vitamin D status and the calcium—phosphate metabolism markers.

## Patients and methods

### The study group

Our study analyzed medical documentation of pediatric patients suspected of calcium and phosphate homeostasis disorders. The analyzed population of patients consisted of neonates, infants and toddlers directed by local general practitioners from entire country for a consultation in the Children's Memorial Health Institute (Warsaw, Poland) in years 1981–2011. Analysis was performed using medical database maintained on an ongoing basis for the scientific and research purposes. The measurements were registered in the database by a physician or nurse after every visit.

The only inclusion criterion for the present study was the availability of 25(OH)D measurement value in a single analysis of a patient‘s sample. 25(OH)D concentration was analyzed in along with other (measured at the same time) parameters of calcium and phosphate homeostasis, including calcium (Ca), phosphates (PO4), and creatinine levels in serum and urine in a 24 h urine sample or in single sample. Alkaline phosphatase activity (ALP) as well as parathormone (PTH) and 1.25(OH)_2_D values were also analyzed if data were available.

Every patient‘s data was analyzed only once and included the first measurement of 25(OH)D concentration, irrespective of the final diagnosis. The study group consisted of children with different calcium and phosphate homeostasis disorders, both congenital and acquired, as well as children who appeared healthy after medical consultation. In total, 3,163 pediatric cases aged 0–3 years (mean age 8 months ± 3) were evaluated.

### Methods

Biochemical parameters were measured in each group using the following methods: total calcium (Ca)—colorimetric assay, Dimension system (Dade Behring), since 2008 photometric assay, Cobas system (Roche); phosphates (PO4)—colorimetric assay, Dimension system (Dade Behring), since 2008 photometric assay, Cobas system (Roche); alkaline phosphatase (ALP)—enzymatic method, Dimension system (Dade Behring), since 2008 colorimetric assay, Cobas system (Roche); 25-hydroxyvitamin D (25(OH)D)—manual protein binding method, since 2005 chemiluminescence immunoassay, Liaison system (DiaSorin); parathormone (PTH) – radioimmunoassay, since 2006 immunoradiometric assay (Cisbio Bioassays); 1,25-dihydroxyvitamin D (1,25(OH)_2_D)—immunoradiometric assay (DiaSource); creatinine—colorimetric assay, Dimension system (Dade Behring), since 2008 enzymatic colorimetric assay, Cobas system (Roche); tubular reabsorption of phosphate (TRP)—calculated indicator. The reference ranges of biochemical parameters used in this study were adapted from the diagnostic laboratory of the Children's Memorial Health Institute as well as from the available reference literature (PO4—in relation to age, TRP, calciuria). For a more detailed analysis of Ca levels in serum, an additional reference range was adopted, which indicated evident hypercalcemia (Ca ≥2.75 mmol/l).

### Statistical analyses

The possible relations between the patient's age, the date of visit, and the 25(OH)D concentration values as well as between the individual biochemical parameters were estimated using statistical correlation models.

The correlation between 25(OH)D concentrations and the year of visit in the clinic was estimated. The studied records were also divided into 9 time periods, each with a comparable number of patients, including the following years: (1981–1999, 2000–2001, 2002–2003, 2004–2005, 2006, 2007, 2008, 2009, 2010–2011). The means ± standard deviation values of 25(OH)D concentrations were calculated for each time period.

The correlation between 25(OH)D concentration values and patients' age was investigated. A studied group was divided into four age-dependent subgroups. Mean values of 25(OH)D and the remaining parameters were calculated. The correlation between 25(OH)D concentration and the patients' age was estimated.

To evaluate possible impact of skin synthesis on vitamin D status, the studied group was divided into four time periods according to the month of the blood sampling in the specific quarter of the year (Q1: January, February, March; Q2: April, May, June; Q3: July, August, September; and Q4: October, November, December). The mean 25(OH)D values were calculated for each quarter. The correlation between 25(OH)D concentration and the date of the visit in each quarter was estimated.

## Results

### General characteristic of the study group

The total number of patients aged 0–3 years with available 25(OH)D concentration values was 3,163 (mean age 8.0 months ± 3.0) (Table 1). The means of the Ca, PO4, ALP, 25(OH)D, PTH, 1,25(OH)_2_D in serum and Ca, creatinine, and TRP in urine were calculated basing on accessible data. All mean values of investigated biochemical parameters were within the reference ranges for a given age (Table 1).

**Table 1:** General characteristics of assessable biochemical parameters in the group of paediatric patients commissioned for calcium—phosphate a vitamin D status evaluations.

**Parameter**	**Number of participants (*n*)**	**Mean (SD)**	**Reference values**
Ca mmol/l	3163	2.54 (0.13)	2.25–2.65 mmol/l
PO_4_ mmol/l	3163	1.90 (0.22)	1–30 days: 1.25–2.50 mmol/l
			1–12 months: 1.15–2.15 mmol/l
			1–3 year: 1.05–1.80 mmol/l
ALP U/l	3160	319.7 (140.9)	< 6 months 120–575 U/l
			6 months-1.5 years 100–550 U/l
25(OH)D ng/ml	3163	37.5 (24.5)	20–50 ng/ml
TRP%	3154	93.8 (6.5)	85–95%
PTH pg/ml	332	23.9 (26.9)	11–62 pg/ml
1,25(OH)_2_D pg/ml	46	62.7 (35.4)	0–2 years-25.1–154.0 pg/ml
			>2–4years-21.8–156.0 pg/ml
Mean age (months)	3163	8.0 (3.0)

### Biochemical characteristics

As shown in Table 2, majority of investigated cases had 25(OH)D concentrations above 20 ng/ml. The mean 25(OH)D concentration was 37.5 ± 24.5 ng/ml. 25(OH)D concentrations of < 10 ng/ml were noted in < 5% of the patients (*n* = 163), values of 10–20 ng/ml in almost 15% (*n* = 465), values of >20–30 ng/ml in 24% of cases (*n* = 756) and 25(OH)D concentrations >30–50 ng/ml in 36% (*n* = 1,136). The maximum measured 25(OH)D value during operation period of 30 years reached 315 ng/ml and was associated with evident hypercalcaemia expressed as Ca = 3.96 mmol/l.

**Table 2:** Presents distribution of the levels of selected biochemical parameters.

**Ca mmol/l**	**%**	***N***
< 2.25	0.5	17
2.25–2.65	85.7	2,711
2.66–2.75	10.5	331
≥2.76	3.4	104
**25(OH)D in serum: min. 0.7 ng/ml, max. 315 ng/ml**			
**25(OH)D ng/ml**	**%**	***N***
< 10	4.5	163
10–20	14.7	465
>20–30	23.9	756
>30–50	35.9	1,136
>50–100	17.8	562
>100	3.2	81
**Alkaline phophatase in serum: min. 36 U/l, max. 5,010 U/l**			
**ALP U/l**	**%**	***N***
< 120	0.5	16
>120–575	96.7	3,056
>575	2.8	88
**Tubular reabsorption of phosphate (TRP) in urine: min. 0.66%, max. 100%**			
**TRP%**	**%**	***N***
< 85%	8.1	254
>85–95%	38.8	1,225
>95%	53.1	1,674

Among the group with severe vitamin D deficiency, defined as 25(OH)D concentration < 10 ng/ml, Ca levels were within the reference range (2.25–2.65 mmol/l) in 83.4% of the patients (*n* = 136), however, two patients were identified with hypocalcaemia (Ca < 2.25 mmol/l) and 25 patients (15.3%) revealed Ca levels indicative of hypercalcaemia (above 2.65 mmol/l). In the subgroup with 25(OH)D values exceeding 100 ng/ml, Ca levels varied between 2.60 and 4.38 mmol/l (on average Ca 2.69 mmol/l; *n* = 80). The percentage of patients with hypocalcaemia (Ca < 2.25 mmol/l) was as low as 0.54% (*n* = 17) and elevated (>2.65 mmol/l) Ca levels were noted in 13.8% of the patients (*n* = 435). The minimal Ca level noted in studied group as a whole was 1.55 mmol/l that coincided with 25(OH)D concentration of 27.8 ng/ml. The highest Ca level was as high as 4.38 mmol/l, with coinciding 25(OH)D concentration of 106.7 ng/ml. Table 3 presents 25(OH)D concentration values in the subgroup of patients with evident hypercalcaemia defined as Ca ≥2.75 mmol/l. The evident hypercalcemia was observed in 3.86% (*n* = 122) cases.

**Table 3:** Distribution of 25(OH)D concentrations among subgroup (*n* = 122) with evident hypercalcaemia (Ca ≥2.75).

**25(OH)D, ng/ml**	***n* = 122**	**mean Ca (SD); mmol/l**
< 10	4	2.80 (0.04)
10–20	20	2.85 (0.15)
>20–30	22	2.84 (0.10)
>30–50	28	2.84 (0.13)
>50–100	32	2.84 (0.14)
>100	16	3.23 (0.57)

It was determined that the prevalence of evident hypercalcaemia was not significantly related to 25(OH)D concentration range, although the lowest number of cases was noted in range < 10 ng/ml. The average Ca level in the group with evident hypercalcaemia was 2.90 mmol/l ± 0.27.

### Analysis of 25(OH)D concentration values according to age

As shown in Table 4, the mean 25(OH)D concentrations in subsequent age groups appeared to be higher than 30 ng/ml.

**Table 4:** Mean 25(OH)D concentrations in subsequent age groups.

**Age (months)**	***n***	**25(OH)D ng/ml (SD)**
0–1	27	27.6 (33.3)
2	68	29.0 (22.7)
3–6	1,112	38.6 (26.5)
7–12	1,489	32.2 (26.6)
13–18	294	37.7 (24.9)
19–36	173	37.1 (19.4)

**Figure 1:**
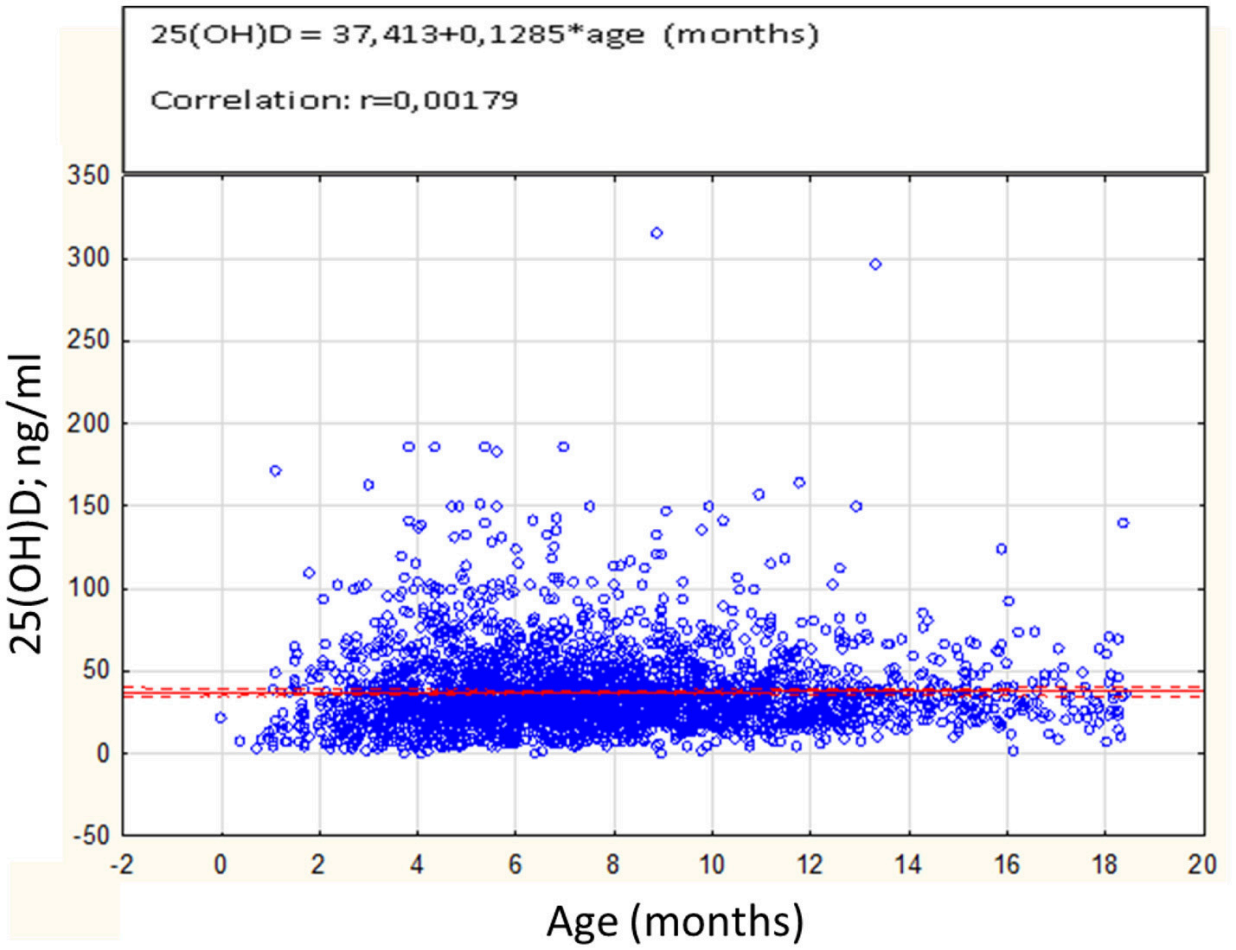
Correlation between 25(OH)D concentrations (ng/ml) and age in months (*n* = 3163).

The highest mean 25(OH)D concentration of 38.6 ng/ml ± 26.5 was observed in infants aged 3–6 months (*n* = 1112). The lowest mean 25(OH)D concentration of 27.6 ng/ml ± 33.3 was noted in neonates aged up to 1 month (*n* = 27). Correlation between the age of investigated cases and 25(OH)D concentrations was not significant (*r* = 0.0018; *p* = 0.921), as shown in Figure 1.

### Analysis of 25(OH)D levels in the respective time periods

Table 5 presents means of 25(OH)D concentration in the specified time periods that corresponded to the patients' visits in the clinic and 25(OH)D assay done in diagnostic unit.

**Table 5:** Mean 25(OH)D concentration values calculated in respective time periods during 30 years of operation of single diagnostic unit.

**Time period (years)**	***n***	**Mean age (months)**	**25(OH)D (SD); ng/ml**
1981–99	305	7	51.8 (38.8)
2000–01	212	7	42.9 (27.4)
2002–03	152	7	48.8 (31.7)
2004–05	325	8	34.8 (19.1)
2006	334	8	36.5 (20.0)
2007	485	8	37.2 (23.1)
2008	457	8	32.9 (18.8)
2009	481	8	36.7 (21.7)
2010–11	412	8	29.0 (13.6)

The highest mean 25(OH)D concentration in analyzed time period was observed in years 1981–1999 (*n* = 305) and reached 51.8 ng/ml ± 38.8. During the following time periods 25(OH)D concentrations significantly trended to decrease (*r* = −0.29; *p* < 0.0001). The lowest mean 25(OH)D concentration value was observed in years 2010–2011 (29.0 ng/ml ± 13.6; *n* = 412).

### 25(OH)D concentrations according to season of the year

25(OH)D concentration values in four quarters of the year are shown in Table 6. Irrespective of analyzed seasons, 25(OH)D concentrations were not significantly different and appeared above 30 ng/ml.

**Table 6:** 25(OH)D concentrations in relation to quarter of the year.

**Quarter of year**	***n***	**mean age (months)**	**25(OH)D (SD); ng/ml**
I	820	7	38.9 (28.5)
II	778	7	37.9 (24.6)
III	747	9	37.5 (24.6)
IV	818	12	35.5 (21.5)

## Discussion

Many reports focused on vitamin D status were published so far. Previously conducted population studies described selected groups, e.g., healthy adults, high-risk groups for vitamin D deficiency—obese or elderly, or were carried out as cross-sectional studies for extensive populations from a single region or country (19, 21–23, 30, 32–34). An increase in interest was concomitant with discussion on vitamin D recommended intakes, reference ranges and potential toxicity risk (24, 25, 31, 47). Most of these reports confirmed that vitamin D deficiency is a serious healthcare problem (22–24, 31, 34).

For many reasons it is not possible to conduct an objective comparison of vitamin D status between modern populations of children and adults, and previous ones from the period of the 1980s or 1990s. Common methods of vitamin D measurement have been used in the laboratory practice since the early 2000s. The methodology was developed simultaneously to discoveries of vitamin D systemic effects. This initiated a dynamic increase in the number of studies evaluating vitamin D status in different groups of patients and/or searching for potential vitamin D—related health benefits. In the last decades of the 20th Century, vitamin D studies were, in general, markedly less often conducted. The 25(OH)D assays were, at that time, based on manual methods that were available only in several reference laboratory facilities, and a scientific surveys included usually a relatively small number of patients.

Our study is based on a representative group of 3,163 pediatric patients admitted to the consultation clinic of calcium and phosphate homeostasis disorders at the Children's Memorial Health Institute over the course of more than 30 years. The first characteristic feature of the study group was a previous suspicion of calcium and phosphate homeostasis disorder, and the other was a broad time spectrum (+30 years), in which vitamin D status was evaluated.

We assumed that in the past, as well as currently, preventive supplementation with cholecalciferol was routinely recommended for newborns and infants up to approx. 18 months of age, resulting in relatively high 25(OH)D concentration values noted during the first visit to the consultation clinic.

The evaluation of the mean values of 25(OH)D in our dataset confirmed above mentioned assumptions and indirectly confirmed effective implementation of rickets-preventive cholecalciferol supplementation among children up to 3 years of age. As expected, the lowest 25(OH)D concentrations were observed in neonates and infants up to the end of a 2nd month after birth, but still the calculated mean values were higher than 20 ng/ml.

The observation of changes in vitamin D status in the studied periods of time revealed a higher 25(OH)D concentrations in the last 2 decades of the 20th Century compared to late 2000s. In analyzed period of time 1981–1999, the average 25(OH)D concentrations appeared as high as 52 ng/ml. In this period the optimal and safe preventive dose of vitamin D was set at 2,500 IU a day, and the therapeutic dose at 4,000 IU a day. Interestingly, at that time a mild forms of nutritional rickets were quite often diagnosed in infants supplemented with vitamin D, however, only in cases who also received in their diets the excessive amounts of phosphates (35, 42). In years 2002–2003, 25(OH)D concentration value in 152 newborns and infants appeared still as relatively high and reached on average 49 ng/ml. In the following years the recommended vitamin D dose was decreased to 1,000 IU/day (in the case of breastfed newborns, even to 400 IU), and most likely resulted in a decrease in average 25(OH)D concentrations observed in the next years. The results of our study represent the first data on 25(OH)D concentration and its trends from a perspective of +30 years of an operation of the single diagnostic unit. The similar idea to review historical data have been already utilized, however, both the analyzed time period and the included populations were different. In a similar study conducted by a single laboratory the retrospective analysis of 1957 blood samples collected from 1909 children and adolescents (age 0–17 years) between year 2009 and 2014 revealed median 25(OH)D concentrations for each year from 2009 to 2014 of: 18, 13, 21, 16, 19, and 15 ng/ml, respectively (48). Further, the two time periods 2009–2012 and 2013–2014 that were analyzed before and after increasing recommendations for vitamin D intake in general population (from 200 to 800 IU per day) did not show any trend for change (17 and 17 ng/ml, respectively) (48).

Further, in another study during the period of 10 years (2007–2017), ~5,000,000 patient samples were tested for 25(OH)D by LC-MS/MS in a single reference laboratory in the USA (49). At the end of summer of 2006, 4.3% of the population being tested had 25(OH)D concentrations < 10 ng/ml. This number increased to 8.5% by the end of winter of 2007. Interestingly, after 10 years, a significantly lower percentage of the population had serum 25(OH)D levels < 10 ng/ml (0.2 and 3.1% post-summer and post-winter of 2017, respectively). Similarly, the percentage of patients with 25(OH)D concentration range of 10–24 ng/ml decreased steadily between 2007 and 2017. By contrast, the percentage of patients with 25(OH)D concentrations (between 25 and 80 ng/ml) increased from 72.5 to 82.4% post-summer and from 60.6 to 72.9% post-winter during the 10 years period (49). The observation that 25(OH)D concentration values in the general US population have increased during the last decade appeared in opposite to estimated trend noted during 30+ years period by our study, and it has important implications. Population based studies and basic science studies exploring the role of vitamin D metabolism in health and disease pathways have raised public awareness about e?ective modes of vitamin D supplementation that appeared more effective in the USA than in Poland. The very similar approach was also utilized in the study of 74,235 serum 25(OH)D results generated under routine conditions between 2015 and 2016 by Italian and Austrian colleagues who were able to document that females had almost 3 ng/ml higher average 25(OH)D concentration than males, which increased significantly with age (50). 37.9 and 28.3% of males and females, respectively, had vitamin D deficiency defined as 25(OH)D concentration of < 20 ng/ml (50).

Our study has some methodological limitations. For example, due to limited number of available data, it was decided to use a possibly controversial method of comparing 20 years-long periods with 1 year-long and 2 years-long periods. Further, the specificity of the analyzed group and the relatively small number of the participants before 1999 are the limitations that should be kept in mind. Nonetheless, our study allowed for estimation of potentially interesting relations, which might be used in further adjustments in the guidelines in order to select the optimal vitamin D supplementation for each age group. The first is the possible relation between skin synthesis (in Poland is restricted to the summer season) and vitamin D status. Taking into account half-life of 25(OH)D (3 weeks), it was assumed that the impact of skin synthesis would be the most evident in Q3 (July-September), and less in Q2 (April-June). However, our study did not reveal any relation between 25(OH)D concentration values and the quarter of the year in which newborns and infants visited the clinic. It was related to a common recommendation to limit the children‘s skin exposure to sunlight. Despite this, 25(OH)D concentrations higher than 30 ng/ml were observed in every quarter of the year suggesting that cholecalciferol supplementation was maintained throughout the year.

The large variety in individual measurements and relatively high values of standard deviation in the analyzed variables can be explained by the heterogeneity of the group, i.e., presence of patients with disorders of calcium and phosphate homeostasis. The study group included patients that after biochemical and clinical evaluation appeared as healthy (without calcium and phosphate homeostasis disorders) as well as those diagnosed with congenital or acquired disorders, including different types of hyperparathyroidism or hypoparathyroidism, patients suspected for idiopathic infantile hypercalcaemia, hypophosphatemic rickets, etc. The relation between obtained results and the type of disease was not a topic of this survey and was extremely difficult to examine basing on assessable database, what is a limitation of this study.

The analysis of correlation between calcaemia and vitamin D status provided interesting observations. It was revealed that in more than 80% of patients with 25(OH)D < 10 ng/ml (severe vitamin D deficiency) Ca levels were within reference range (2.25–2.65 mmol/l). On the other hand, in subgroup with hypocalcaemia (Ca < 2.25 mmol/l) severe vitamin D deficiency was not a serious problem. Only 11% of hypocalcaemic patients revealed 25(OH)D concentration values lower than 20 ng/ml, whereas 39% of these patients demonstrated 25(OH)D below 30 ng/ml. In the analyzed subgroup with evident hypercalcaemia (Ca ≥2.75 mmol/l) both very low and high 25(OH)D concentrations were observed, and what seems interesting and potentially meaningful, there was no correlation between the severity of hypercalcaemia and the 25(OH)D concentration values. Moreover, a number of cases with evident hypercalcaemia had 25(OH)D concentrations lower than 10 ng/ml or higher than 50 ng/ml. Using an appropriate set of biochemical and clinical parameters it was possible to identify patients with other characteristic phenotypes of calcium and phosphate homeostasis disorders, e.g., features of hyperparathyroidism.

Another important limitation of our study is related to methodological changes in 25(OH)D measurement over the analyzed time period. In our laboratory, until year 2005, 25(OH)D measurements were carried out using manual protein binding method, and later with the use of automatic Liaison system based on chemiluminescence immunoassay. However, it is worth mentioning that both methods were compared and validated with GLPs, and the high precision of 25(OH)D measurements was confirmed with a DEQAS international quality certificate. Unfortunately, it was not possible to include the more recent 25(OH)D data to indicate possible trends and its changes over the last 15 years, what is limitation of our attempts. Nonetheless, the recent studies conducted in Poland have already uncovered vitamin D status in newborns, infants and toddlers, and confirmed the problem of low 25(OH)D concentrations in the second decade of XXI century. The MAVID RCT study from the Warsaw city area revealed that < 10% of mother-newborn pairs had 25(OH)D >30 ng/ml and the median 25(OH)D concentration in newborns was as low as 15 ng/ml (51). In the other study, vitamin D status of newborns and infants was shown to get improved, reaching the 25(OH)D concentration value of 43 ng/ml ± 20 at the 6 months of life (52). Unfortunately, at the 12th month of life, in the same group of infants 25(OH)D concentration values significantly decreased to the mean of 29 ng/ml (*p* < 0.0001), despite that the total vitamin D intake from diet and supplements was close to 1,000 IU/d and was not significantly different at both the 6th and the 12th month of their life. Interestingly, the observed 25(OH)D concentration decrease was related to reduced total vitamin D intake expressed in in international units per kilogram body weight that decreased significantly from 143 IU/kg body weight at the 6th month to 93 IU/kg body weight at the 12th month (*p* < 0.0001) (52).

Further, it was evidenced that in Poland vitamin D deficiency (< 20 ng/ml) affects about 17% of children aged 2–3 years (53), in ~35% of children aged 3–4 years (54) and even in 87% of older children and adolescents, depending on the season of the year and the age group (55).

Nonetheless, more research focused on vitamin D and its metabolites is still required and our study results, due to its limitations, provided only the estimation of unfavorable trends of lowering 25(OH)D concentrations in the youngest kids during the period of 30 years. While a dynamic growth in prospective studies involving children and adults can be predicted, the possibility of the retrospective assessment of data from before 2000 is very limited. The mentioned situation justified analyzing and describing available data from that period, and contributed to the preparation of this study, which also fits in the stream of reports and publications focused on the risk of vitamin D deficiency. What is worth mentioning is that even in that group of children aged 0–36 months, almost 20% revealed vitamin D deficiency expressed as 25(OH)D concentrations below 20 ng/ml. The assessment of the extensive pediatric patient population covering 3 decades can, in our opinion, complement the knowledge of the past and current state and trends in the vitamin D status in very young children residing in Poland.

## Ethics statement

This study was carried out in accordance to standard diagnostic procedures. The study was conducted basing on medical records of pediatric patients admitted to the consultation clinic of calcium and phosphate homeostasis disorders. Ethics Committee approval was not needed to analyse database.

## Author contributions

All authors listed have made a substantial, direct and intellectual contribution to the work, and approved it for publication.

### Conflict of interest statement

The authors declare that the research was conducted in the absence of any commercial or financial relationships that could be construed as a potential conflict of interest.
